# Mapping Yellow fever epidemics as a potential indicator of the historical range of *Aedes aegypti* in the United States

**DOI:** 10.1590/0074-02760220306

**Published:** 2022-04-06

**Authors:** Nicole S Fijman, Donald A Yee

**Affiliations:** 1 University of Southern Mississippi, School of Biological, Environmental, & Earth Sciences, Hattiesburg, Mississippi, USA

**Keywords:** Aedes aegypti, arbovirus, Yellow fever, historical range

## Abstract

**BACKGROUND:**

Yellow fever (YF) plagued the United States from the 1690s until 1905, resulting in thousands of deaths. Within the US, *Aedes aegypti* is the only YF vector and almost no data exists for the location of this species prior to the early 1900s.

**OBJECTIVES:**

To determine the historical range of *Ae. aegypti* we examined the occurrence of YF epidemics across time and space. We hypothesized that historically *Ae. aegypti* was driven by human population density, like its contemporary range suggests.

**METHODS:**

To test this hypothesis, we compiled a list of YF cases in the US, human population density, location, and the number of people infected. This data was mapped using ArcGIS and was analyzed using linear regression models to determine the relationship among variables.

**FINDINGS:**

The historic range was generally south of 40º latitude, from Texas in the west to Florida in the east, with concentrations along major waterways like the Mississippi River. Infected individuals and human population density were strongly correlated across the whole dataset as well as by decade.

**MAIN CONCLUSIONS:**

Although other factors likely affected the range of *Ae. aegypti*, we found that human population density was related to the number of people infected with historic YF infections.

Yellow fever (YF) was an important infectious disease throughout the Americas in the 17th, 18th, and 19th centuries.^1^ Today, estimates show that annually around 200,000 people world-wide are infected with YF, mostly concentrated in areas with low vaccination rates, despite the vaccine being highly effective and safe.^2^ This virus most likely originated in Africa and was brought to the Americas through the slave trade spanning the 15th to 17th centuries.^3^ The disease was likely brought over in conjunction with the aquatic stages of the mosquito host, *Aedes aegypti*, the main historic vector of YF, aboard ships from Europe via Africa.^3^ Due to the large number of ships that followed these trade routes between Europe, Africa, and the West Indies, it is likely that *Ae. aegypti* entered the Americas on several separate occasions.^3^ People infected with YF were also brought to the Americas on slave ships, who harbored the virus and allowed transmission to new populations in the Americas.^5^ In tropical and subtropical areas, *Ae. aegypti* likely colonized and was able to overwinter; however, in more northern areas like New York and Philadelphia, reintroduction via trade ships was probably necessary.^4^ YF has been a part of United States history since it was first introduced in 1691 in Boston, Massachusetts.^5^ The fear of YF led to many different names for the disease including Yellow Jack, the Saffron Scourge, Bronze John, and the Yellow Tyrant of the Tropics.^6^ ^,^ ^7^ Although epidemics began in the 17th century, it was not until 1900 that Major Walter Reed, Dr Carlos Finlay, and their colleagues discovered that mosquitoes were responsible for transmission among humans.^8^ Before this discovery, prevention methods were generally misdirected and ineffective at preventing the spread of YF. Moreover, until mosquitoes were implicated as disease vectors, there was no thought to document the distribution and range of any mosquito species. This fact complicates efforts to understand the historical distribution of *Ae. aegypti*.

The YF virus belongs to the genus Flavivirus (family Flaviviridae), a genus of arboviruses that is transmitted to a vertebrate host through an arthropod vector.^8^ YF has two distinct transmission cycles. Sylvatic YF virus occurs in tropical forests and circulates between non-human primates and mosquito populations, whereas urban YF circulates in densely populated centers, where humans are the most abundant primate.^1^ For the urban cycle, the virus multiplies in the infected mosquito, is stored in the salivary glands, and is transmitted to humans through the bite of an adult female.^1^ Once bitten, the incubation period in humans is 3-6 days.^2^ Although YF globally is transmitted by other species of mosquitoes, in the US *Ae. aegypti* is the only vector.^9^ In humans, YF causes fever, liver dysfunction, renal failure, hemorrhaging, and circulatory collapse, which can lead to death in five to six days after the virus has incubated in the host.^1^ Today, most people that contract YF are asymptomatic; however, of the people that develop severe reactions, the fatality rate is between 30-60%.^10^

*Aedes aegypti* uses containers for larval development, preferring to oviposit in small water-holding containers.^11^ Although there is not much research on the historic breeding sites for mosquito larvae, homes typically had cisterns with standing water that might have been used for oviposition, and city sanitation laws were not in place to clean up standing water.^6^ *Ae. aegypti* prefer to take blood meals from humans over other hosts.^12^ ^,^ ^13^ ^,^ ^14^ Scott et al.^14^ found that when given a choice, *Ae. aegypti* prefers human blood compared to dogs, chickens, bovines, rats, and cats in Thailand. Harrington et al.^13^ found that the higher concentration of isoleucine in human blood gives *Ae. aegypti* increased fitness, which may explain this preferential host feeding. A human preference for blood may drive this mosquito species to live in areas of high human concentration. *Ae. aegypti* takes multiple blood meals during one reproductive cycle, increasing the amount of people exposed to an individual mosquito,^12^ and thus making it more efficient at passing on human pathogens like YF.

In the US, there have been approximately 88 major epidemics of YF recorded between 1691 and 1905, killing an estimated 100,000 to 150,000 people.^5^ Although the disease began in port cities on the East Coast, it spread to the South, partly due to the railway system, where it affected urban areas.^1^ The last major occurrence in America was in 1905 in New Orleans, Louisiana, where 497 people died.^5^ Because YF was exclusively transmitted by *Ae. aegypti* in the US, the historic range can be inferred by using epidemics as a subset of the mosquito population.^5^ The drawbacks to this method is that there likely are areas where the mosquito persisted, however the virus was not present, although to mitigate this effect we used a larger time period. Thus, it can be assumed that YF incidence reflects a conservative estimate of the presence of *Ae. aegypti* in that location at that time.

Although a major occurrence of YF has not occurred in the US in more than 100 years, *Ae. aegypti* still threatens global health, as this species can transmit other arboviruses like dengue, Chikungunya, and Zika.^2^ Not much is known about the factors that influenced the historical establishment and spread of *Ae. aegypti*, which is now currently found throughout tropical and subtropical regions. Examining the historical occurrences of YF and human population density could determine whether this has always been a driving factor in the range of *Ae. aegypti* or if it has evolved during its establishment and spread through the Americas. Given its proclivity for biting humans, we hypothesized that human population density was historically an important factor in the range of this mosquito, with this species being concentrated in larger urban centers like cities, where it was more likely to encounter humans. We predicted that historical occurrences of YF would be correlated with areas of high human population density given the association between this mosquito and the preference for human blood meals. To test this prediction, we used the historical record of YF cases as a proxy for occurrence of *Ae. aegypti*, as the disease has no other means of transmission outside of the mosquito. Due to the lack of knowledge of the mosquito ecology historically, YF occurrences are one of the few ways to learn more about where the range of *Ae. aegypti* and the factors that could affect the spread of YF.

## MATERIALS AND METHODS

The purpose of this study was to determine if human population density was predictive of the historical range of *Ae. aegypti* using YF occurrences as a proxy for the presence of this mosquito. The full range of *Ae. aegypti* is unable to be captured through this method because not all individual mosquitoes were infected with YF and therefore cannot be represented through this study. Although there are more variables that likely affect the spread of YF (e.g., temperature), we focused on human population density, due to the limited availability of other data from that time.

Data collection involved several steps. The first step was to compile recorded occurrences of YF, beginning in 1870 through 1905. This period was chosen to correspond to the establishment of nation-wide public health systems. Prior to the 1870s, data from the US on disease outbreaks was sporadic and uncoordinated. An occurrence, in this study, is defined as any location where at least one record of YF was recorded for one year. For every recorded incident of YF, we documented, where possible, the year of occurrence, the city, county, state, the number of people infected, and the population of the location when this occurrence occurred. These records were obtained through the primary literature, epidemic reports, US census data, and other sources.^2^ ^,^ ^5^ ^,^ ^6^ ^,^ ^7^ ^,^ ^9^ ^,^ ^15^ ^-^ ^20^ Overall, 424 occurrences of YF were recorded for this period. Of these only 69 had direct city level population density data for the year of the outbreak. To find another source for the population density, we used the US census data. Because census data are taken only every ten years, we grouped epidemics by decades and used the census data as an estimate of the population during the epidemic. For example, every YF epidemic from 1870-1879 used the human population from the 1870 census. The census recorded population by county, while most epidemics were recorded by city. To determine if the census data by county was an accurate representation of the population, a simple linear regression model was conducted to compare the population of the city to the population of the county from census data. We used 69 epidemics and found that the variables had a significant positive linear correlation (p < 0.001, R^2^ = 0.2673). Thus, the county population size could be used to represent the population for cities that occurred within them.

Second, after this data was compiled, it was incorporated into ArcGIS 10.6.^21^ The data was mapped to assess visually the epidemics in ten-year periods to correlate with the census data. Each map showed the number of people infected represented by graduated symbols, and graduated shading represented the population density, with darker colors reflecting more densely populated locations.

Third, human population density and infected individuals were analyzed to determine if there was a relationship between these variables. Relationships were assessed using simple correlation analysis and generalized linear models. The first analysis performed was to determine if human population density was related to the number of infected individuals. To do this, both human population density from county level census data and city level data were used. First, we identified the 69 epidemics that had city data from the year of the epidemic, and then generalized linear regression models with a Poisson distribution were used to determine whether city population could predict the number of people infected. Next, we ran the same analysis with the number of people infected and the population of the county based on census data. These two were used to ascertain if there was a relationship between human population density and infected individuals, and whether this relationship was more apparent with city level data. In the second analysis, we ran another set of generalized linear models using a Poisson distribution that predicted infected individuals at each decade from human populations. Four generalized linear models were performed, one for each decade (1870s, 1880s, 1890s, and 1900s). All analyses were preformed using R software (R Core Team, packages = vegan and BiodiversityR).

## RESULTS

**Fig. 1:**
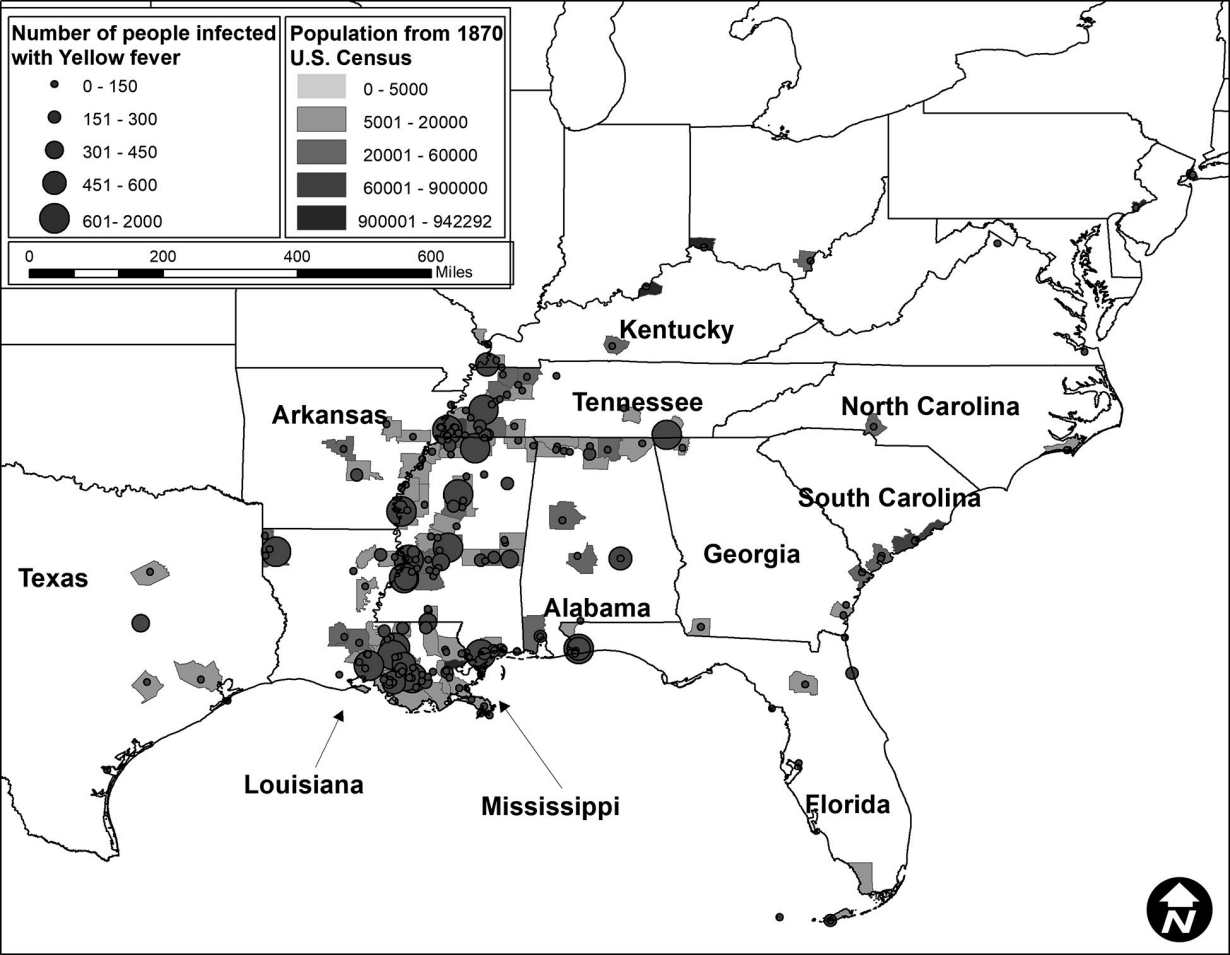
location of Yellow fever (YF) occurrences and the number of infected humans with YF from 1870 to 1879. The counties are colored based on the human population density, where darker colors represent locations with more people. This decade has the most occurrences of YF, especially in 1878, when a wide spread epidemic of YF affected places as far north as Ohio. Sources Esri, National Atlas of the United States, US Geological Survey.

**Fig. 2:**
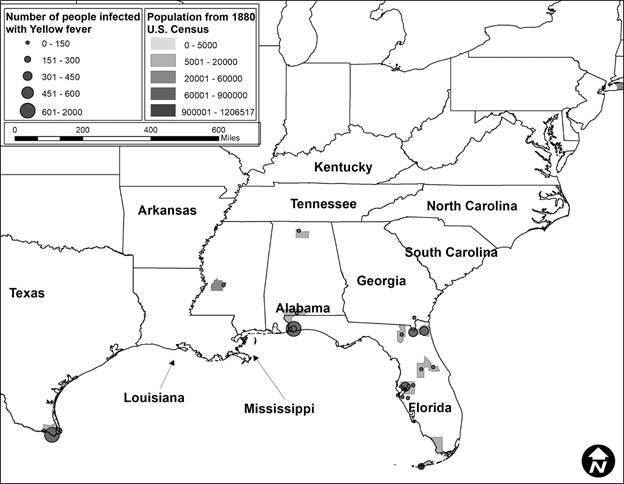
location of the Yellow fever (YF) occurrence and the number of infected humans with YF from 1880 to 1889. The counties are colored based on the human population density, where darker colors represent locations with more people. Note how many fewer occurrences during this time period compared to other decades and the lack of occurrences in Louisiana.

**Fig. 3:**
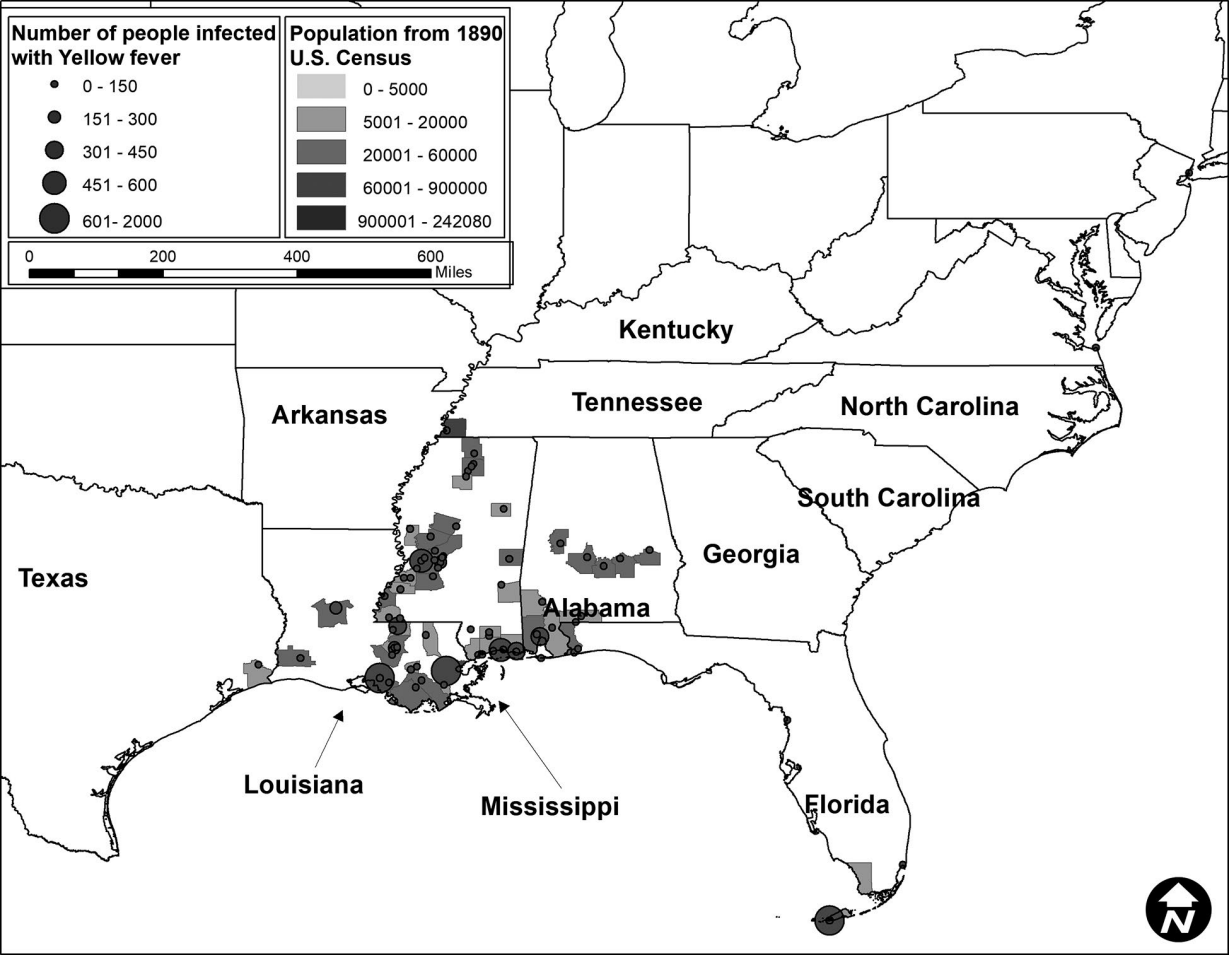
location of Yellow fever (YF) occurrences and the number of infected humans with YF from 1890 to 1899. The counties are colored based on the human population density, where darker colors represent locations with more people. Note the clustering of epidemics in southern states (Texas, Louisiana, Mississippi, Alabama, and Florida). Many of the epidemics are found in locations with higher population densities, especially in Louisiana, Mississippi, and Alabama.

**Fig. 4:**
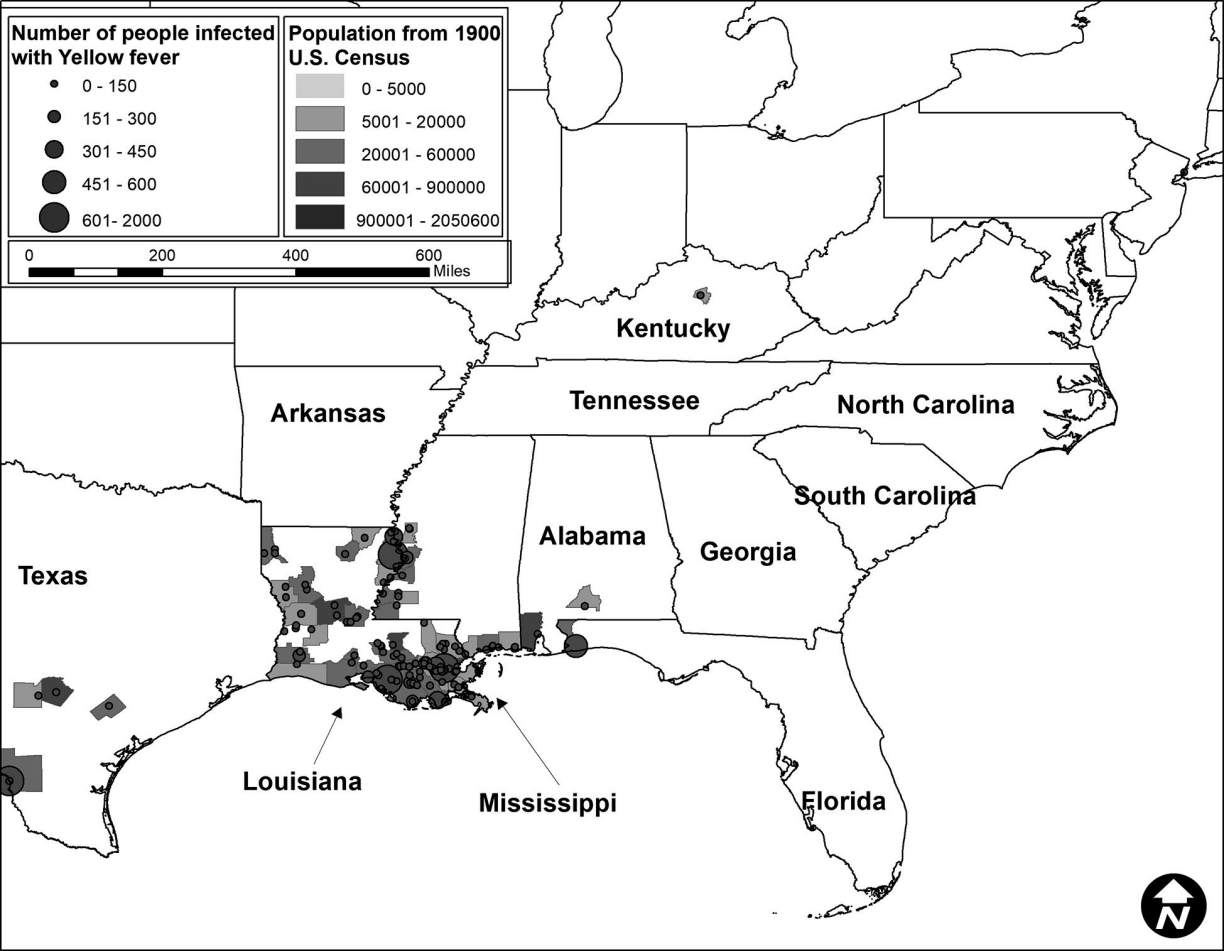
location of Yellow fever (YF) occurrences and the number of infected humans with YF from 1900 to 1905. The counties are colored based on the human population density, where darker colors represent locations with more people. Note the epidemics are along the entire coast of Louisiana and Mississippi. There are very few recorded epidemics outside of these states, except in Texas and Alabama. The last epidemic occurred in Mobile, Alabama in 1905.

**Fig. 5:**
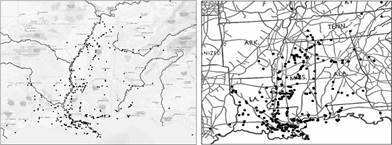
Yellow fever (YF) epidemics from 1870 to 1905 overlaid on maps of the major water ways (black lines, left panel) and railroads (gray lines, right panel) in 1890. Each black point represents the location of an occurrence. Note the clustering of epidemics along both transportation routes. Transportation routes were placed on separate panels to facilitate clarity.

**Fig. 6:**
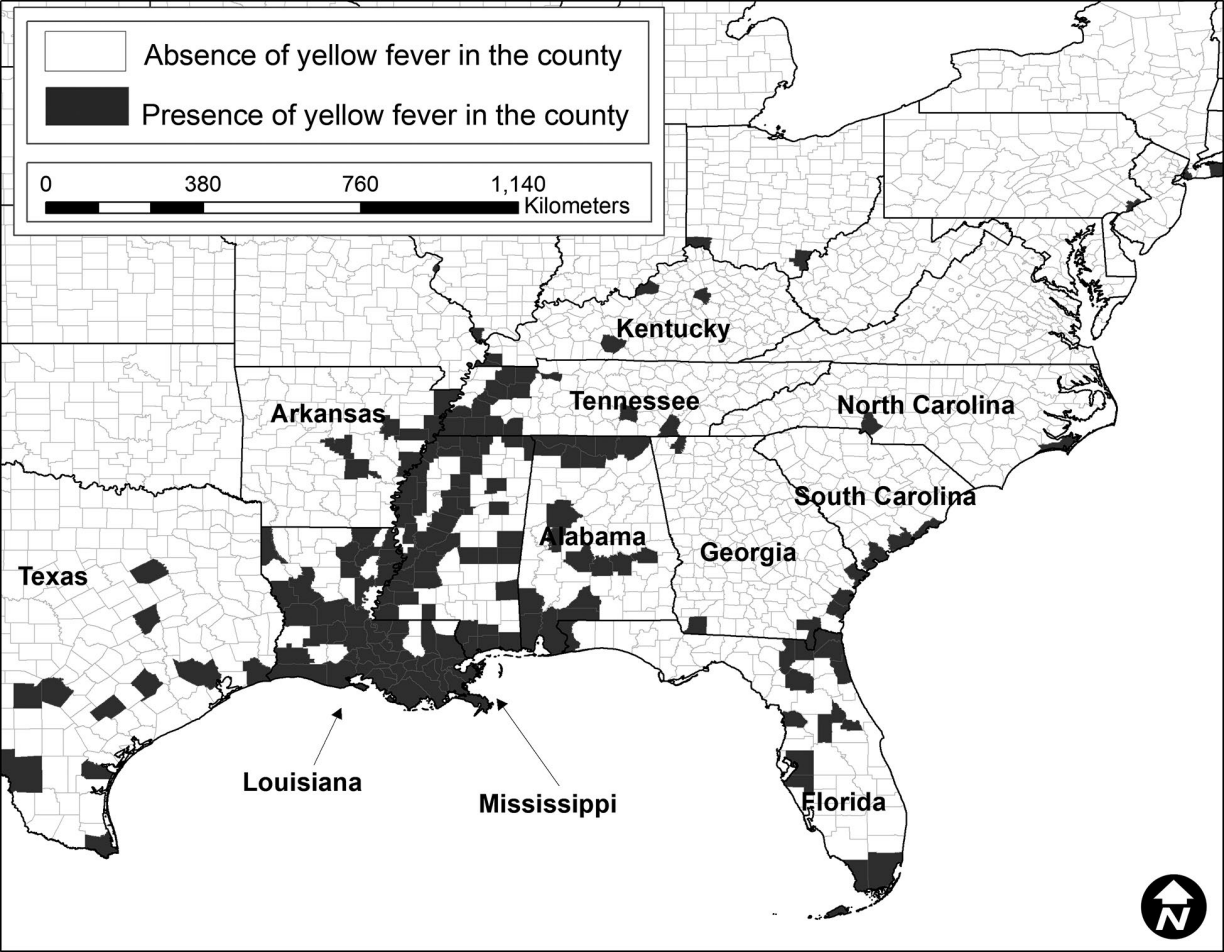
counties with reported cases of Yellow fever (YF) during 1870 - 1905. This map may serve as a historical range for *Aedes aegypti* mosquito because YF virus was only spread by this mosquito.

In total, 424 YF occurrences were identified in the US from 1870 to 1905. The number of occurrences varied by decade. In 1870 to 1879 (Fig. 1), there were 248 occurrences, in 1880 to 1889 (Fig. 2) there were 28, in 1890 to 1899 (Fig. 3) there were 92 occurrences, and in 1900 to 1905 (Fig. 4) there were 137 occurrences. A single composite map was produced that illustrated every location of YF near the Mississippi River to represent visually the correlation between the river and the spread of the epidemics (Fig. 5). Finally, our data allowed us to show the hypothesized historic range of *Ae. aegypti* in the US, with all counties where YF, and thus *Ae. aegypti*, was present from 1870 - 1905 (Fig. 6).

The first analysis determined whether human population density could be used to predict the number of infected individuals. Although there was a relationship between census data and the number of people infected with YF, the city population size had a higher correlation with the number of infected individuals. Next, generalized linear models were run on the same dataset using the human population as the predictor variable and infected individuals as the response variable. Here, both census data and city data were significant (F_1,67_ = 18.97, p < 0.001, R^2^ = 0.21, mean infected population size = 646.1 ± 16,606.0), showing that both population data sets can be used to predict infected individuals.

For the second analysis, human population density based on the census data significantly and positively affected infection rates of YF (F_1,67_ = 42.94, p < 0.001, R^2^ = 0.38, mean infected population size 8,728.1 ± 218,524.2). Because of the significant relationship between the number of infected individuals and both city and census human population density and across each decade, these results indicate a historic relationship between human population density and the range of *Ae. aegypti*.

## DISCUSSION

The purpose of this study was to determine if YF virus occurrence and historic human population sizes were predictive of the range of *Ae. aegypti* mosquito by using YF epidemics to represent a subset of the mosquito population. We show that there is a correlation between human population and the number of infected individuals. When epidemics were analyzed by decade, each decade showed a significant and positive correlation between human population density and the number of infections. This information supports the hypothesis that cities with higher population densities had higher rates of YF occurrence. This was true even though the number of epidemics varied greatly, from a low of 28 occurrences between 1880 and 1889, to a high of 248 occurrences from 1870 to 1879. However, although we can assume that every location with an occurrence of YF had *Ae. aegypti* present, we have no way of estimating their actual abundance.

A historic range map of *Ae. aegypti* from 1870 - 1905 was created using counties that had YF virus present (Fig. 5). This map shows the most accurate range of the mosquito using historical occurrence of YF as a proxy. From this map, there are several conclusions that can be drawn. First, the concentration of counties with occurrences of YF was in the south (Figs 1-4). This is consistent with the fact that *Ae. aegypti* is a species that favors tropical to subtropical areas where overwintering is possible.^1^ Second, in addition to the concentration in southern cities, port cities in the northern cities along railways or waterways, often had individuals infected with YF (and thus *Ae. aegypti*) (Fig. 5). This illustrates a likely pattern of spread for the disease because it almost always originated at or near a port and then would likely spread inland along transportation routes like railways.

Although this research focused exclusively on human population as a factor of *Ae. aegypti* presence, there may have been many factors that were not analyzed, including climate or other human patterns affecting disease spread.^1^ Inclusion of these factors could have led to stronger relationships between human populations and YF occurrences. Climate factors, including temperature and precipitation, affect the range of *Ae. aegypti*, which likely affects the spread of YF.^1^ ^,^ ^22^ In general, mosquito abundance is affected by annual temperature and rainfall.^23^ One of the climate factors that has been shown to affect mosquito abundance is an El Niño, which can lead to increased precipitation, likely leading to ideal breeding conditions for *Ae. aegypti*;^16^ ^,^ ^24^ most major YF occurrences have been linked to El Niño events.^16^ The epidemic of 1878, which greatly affected the Mississippi Valley and resulted in over 20,000 deaths was during an El Niño year.^9^ ^,^ ^16^ Philadelphia, the site of early epidemics, including the epidemic of 1793 was also during a strong El Niño year and resulted in approximately 5,000 deaths.^24^ This epidemic was the worst the country had seen at this time, sparking panic among residents of the then US capitol.^25^ Thus, these weather anomalies caused by El Niño could also have affected the variance and abundance in occurrences of YF in the US. Socioeconomic factors were not analyzed in this study, even though they likely play an important role in the expansion of *Ae. aegypti* range and the spread of YF. People of higher socio-economic status were likely to have technology in place that could prevent mosquitos from entering their homes, and they likely had less standing water and trash receptacles that could be used as breeding sites.

In this study, the effect of human movement as a measure of globalization was not explored as an avenue of YF spread. Historically, cities located on the Gulf, on the Mississippi River, or near a railroad station had higher rates of YF.^16^ ^,^ ^17^ As humans became more connected through travel, viruses spread more frequently, a phenomenon that has been studied throughout history.^26^ Records from the period can be somewhat skewed based on access to health care and race relations, which were especially tumultuous after the US Civil War (1861-1865), a period right before the epidemics studied in this work. Moreover, the actual number of people infected with YF is likely higher than what historical records show, especially if African Americans and Native Americans were undercounted, which given the years before and after the Civil War, was a likely situation. In addition, although we found a strong link between human population density and YF, it is likely that many instances of the disease went unreported, owing to a lack of communication and record keeping in the pre-industrial age.

During the mid-19th century railways helped to connect America in a way that had never been available before.^16^ In this case, railways allowed people infected with YF virus to travel farther and spread the disease to areas away from the initial source of infection.^6^ ^,^ ^17^ This may have hastened the spread of YF to smaller communities that would not normally have been exposed to infected individuals. Starting in the mid-1850s when New Orleans and Memphis were connected by railroads, YF became common in both cities.^9^ Railroads are considered the reason that *Ae. aegypti* was able to spread north to Memphis, Tennessee resulting in thousands of deaths in the region.^9^ Railroads were frequently used by infected individuals to flee cities where YF occurred, which likely brought infected people to formally disease-free areas as people sought refuge from the disease.^6^

Although railroads allowed YF to spread once it was in the US, the international and national shipping industry allowed YF to cross the Atlantic on an annual basis.^3^ ^,^ ^9^ YF was introduced through port cities bringing people infected with YF first from Africa in the slave trade and later from the Caribbean.^3^ ^,^ ^9^ One of the factors that predicted YF in the US was the rate of YF in the Caribbean.^7^ Major port cities along the Gulf of Mexico like New Orleans, Louisiana, and Mobile, Alabama, and river port cities like Memphis, Tennessee had YF present in almost every decade analyzed (Figs 1-4). The steamboat shipping industry on the Mississippi River also allowed YF virus more access to port cities, both large and small (Fig. 5). In Philadelphia, it is believed that YF and *Ae. aegypti* were introduced multiple times through the shipping industry because the mosquito could not survive the winter.^3^ This ability to spread via man-made vessels allowed *Ae. aegypti* to become a worldwide invasive species.^4^ Although transportation of YF and *Ae. aegypti* was not the focus of this paper, it clearly played a role in the transmission of the virus (Figs 5-6) and likely plays a role in the historic outbreaks of the virus in the US.

Historical events, including legislation efforts to create federal health aid, may also have affected the transmission of YF. The National Board of Health was created in 1879, after the widespread epidemic in 1878 across the Mid-South. At the time, states had their own Boards of Health that would operate independently of the federal government. This sometimes resulted in biased actions that benefited the economy of the state rather than the health of the people. The National Board of Health began passing legislation to prevent future occurrences of YF by enacting the Quarantine Act of 1879 to prevent infectious diseases from entering the country and general sanitation laws.^17^ Most of the legislation, however, was ignored due to the cost and inconvenience, especially in New Orleans, which did not improve its sanitary conditions.^18^ Because the mechanisms of transmission of YF were unknown, the Quarantine Act of 1879 did not stop the influx of the virus, but once sanitation practices were adopted, these helped to reduce *Ae. aegypti* larval habitats.^15^ This may explain the relatively fewer epidemics after 1878, mostly via the quarantine of sick individuals and removal of larval habitats of *Ae. aegypti* on ships.

Knowledge about the historical range of *Ae. aegypti* may help us understand the contemporary range of this species and the spread to new places as globalization increases and climate change continues to affect habitats and abiotic factors. The contemporary range of *Ae. aegypti* was affected by human population density, along with temperature and precipitation.^4^ ^,^ ^27^ As the climate changes, mosquito habitats will likely expand to include new areas where temperature and rainfall can support populations where they previously have not existed.^28^ Specifically, in the US, the range of *Ae. aegypti* will likely get broader, resulting in new areas exposed to this mosquito and the pathogens it transmits.^28^ As the world becomes more connected, viruses are more likely to spread to new locations.^26^ Urbanization, trade routes, and travel all make the world a smaller place for disease spread by connecting humans that were historically separated.^26^ Through the study of YF in the US historically, we can see examples of how climate change, urbanization, and trade routes affected the transmission. All these factors still play a role today as *Ae. aegypti*, a vector of dengue, chikungunya, Zika, and YF, continues to spread disease globally.
